# Supplementary material for: CD28 engagement inhibits CD73-mediated regulatory activity of CD8+ T cells
Source: Commun Biol. 2021 May 19;4:595. doi: 10.1038/s42003-021-02119-9 (PMC8134507; doi:10.1038/s42003-021-02119-9)
Supplement: Supplementary file 2 — Supplementary Information [file 42003_2021_2119_MOESM2_ESM.pdf]

**Supplementary information**

**Title:**

CD28 engagement inhibits CD73-mediated regulatory activity of $CD8^+$ T cells

**Short Title :**

CD28 costimulation suppress CD73 expression

**<u>Authors</u>:**

Yo-Ping Lai[1], Lu-Cheng Kuo[1], Been-Ren Lin[2], Hung-Ju Lin[1], Chih-Yu Lin[3], Yi-Ting Chen[4], Pei-Wen Hsiao[3], Huan-Tsung Chang[5], Patrick Chow-In Ko[6], Hsiao-Chin Chen[7], Hsiang-Yu Chang[5], Jean Lu[8,9,10], Hong-Nerng Ho[11,12], Betty A. Wu-Hsieh[12], John T. Kung[4], Shu-Ching Chen[7]*

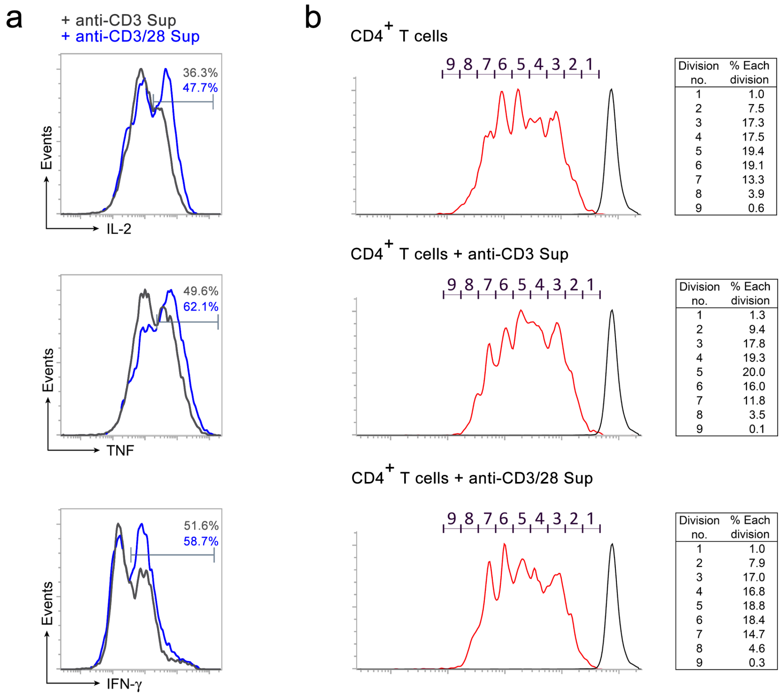


**Supplementary Figure 1. The effects of suppressive CD8$^+$ T cells on CD4$^+$ T cell cytokine production and proliferation.** CFSE-labeled naïve CD4$^+$ T cells were activated by anti-CD3/28 antibodies in the presence of supernatants (50% volumn) from anti-CD3- or anti-CD3/28 antibodies-stimulated CD28WTCD8$^+$ T cell cultures. **a** At 24 h after stimulation, CD4$^+$ T cell production of TNF, IFN-γ and IL-2 was measured by intracellular staining and analyzed by flow cytometry. Percentages of cytokine-producing populations are shown. *Black*, addition of anti-CD3 Sup. *Blue*, addition of anti-CD3/28 Sup. **b** As in **a**, at 64 h after stimulation, cells were harvested and cell division was analyzed by flow cytometry. *Black*, cells at 0 h. *Red*, cells at 64 h

after stimulation. Box on the right, percentage of cells in each division. The data represents one of the three independent experiments.

a

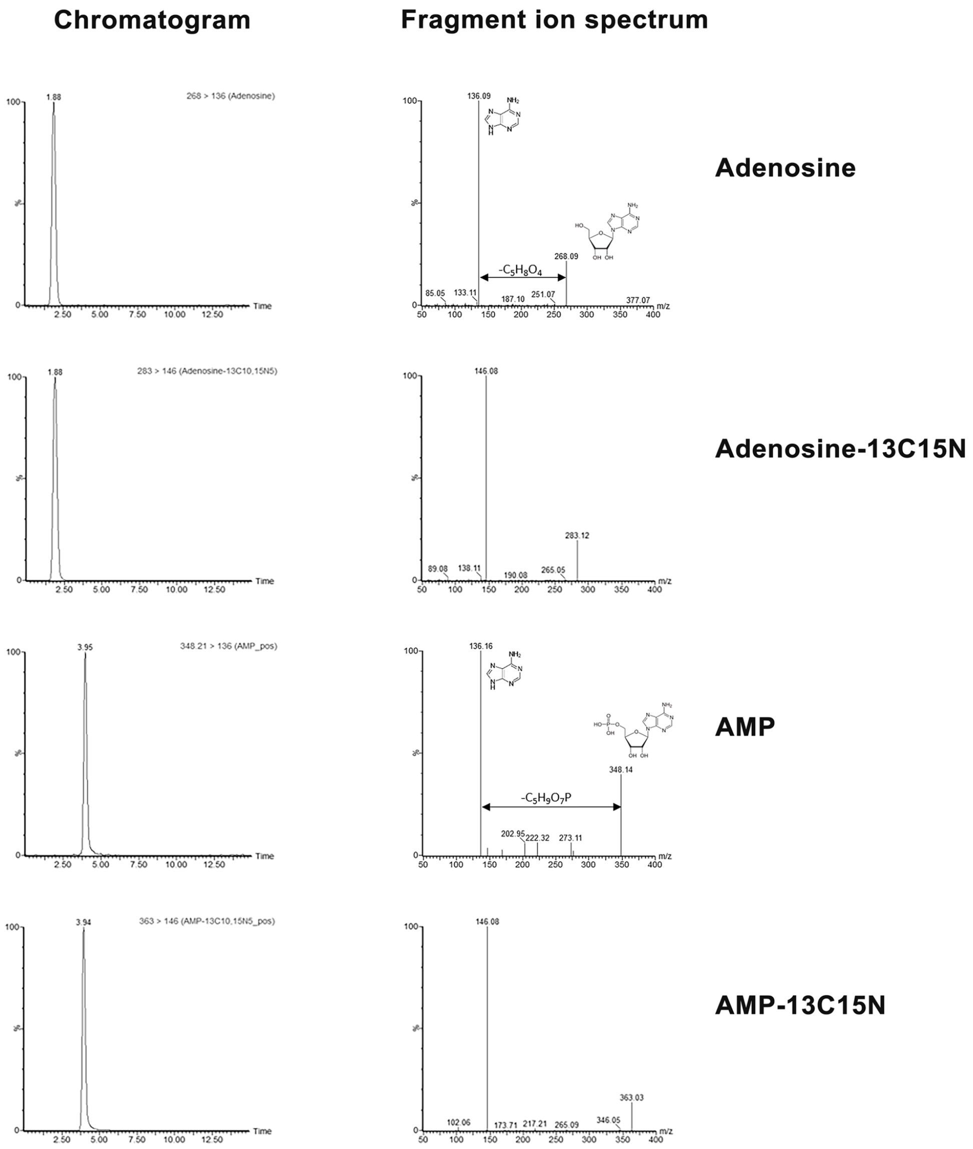
Chromatogram
Fragment ion spectrum
268 > 136 (Adenosine)
283 > 146 (Adenosine-13C10,15N5)
348.21 > 136 (AMP_pos)
363 > 146 (AMP-13C10,15N5_pos)
Adenosine
Adenosine-13C15N
AMP
AMP-13C15N
Time
m/z

b

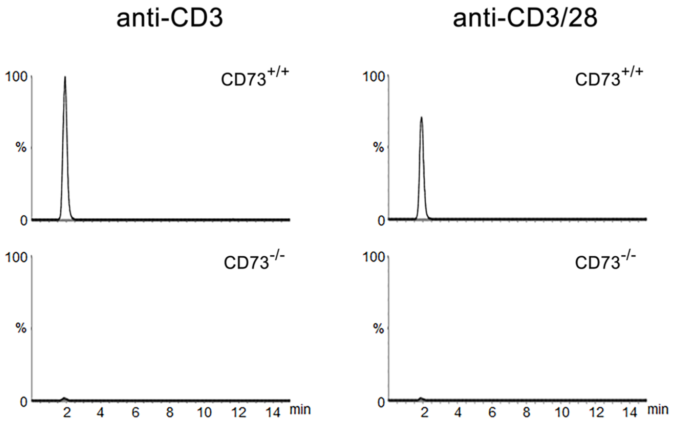
anti-CD3
anti-CD3/28
CD73+/+
CD73-/-
100
%
0
min

c

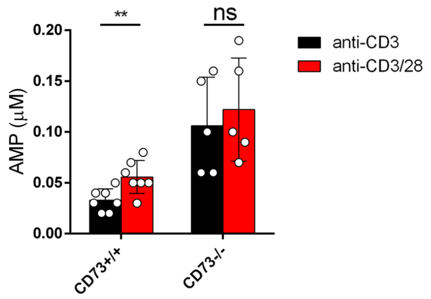


d

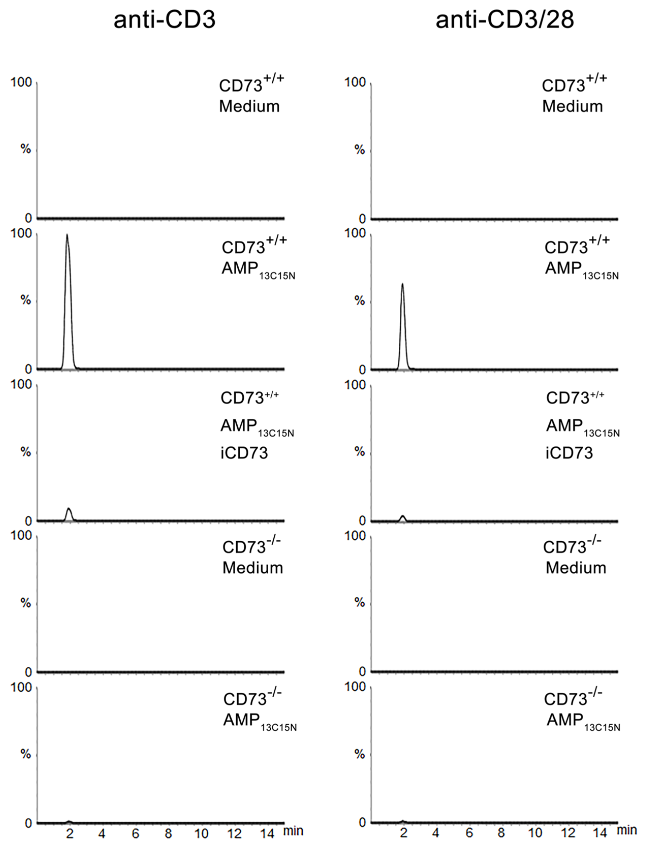


e

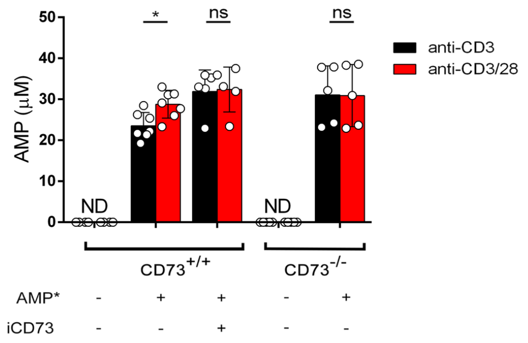


**Supplementary Figure 2. Measurement of adenosine and AMP by UPLC-MS/MS.**

**a** Detection of adenosine and AMP in culture supernatant by UPLC-MS/MS. *Left*,

Representative MRM Chromatograms of adenosine, $^{13}C10,^{15}N5$ adenosine, AMP and $^{13}C10,^{15}N5$-AMP. The MS/MS transitions were monitored using multiple reaction monitoring (MRM) mode for adenosine (m/z, 268 > 136), $^{13}C10,^{15}N5$ adenosine (m/z, 283 > 146), AMP (m/z, 348 > 136) and $^{13}C10,^{15}N5$ AMP (m/z, 363 > 146). *Right*, MS/MS spectrum of adenosine (m/z 268), $^{13}C10,^{15}N5$-adenosine (m/s 283), AMP (m/z 348) and $^{13}C10,^{15}N5$-AMP (m/z 363). **b** Endogenous generation of adenosine in culture supernatants of CD28WTCD73WTCD8$^{+}$ and CD28WTCD73KOCD8$^{+}$ T cells after stimulation by anti-CD3 or anti-CD3/28 antibodies was quantified at the indicated retention time (x-axis). The quantity of adenosine in the supernatant of CD28WTCD73WTCD8$^{+}$ T cell culture stimulated with anti-CD3 antibody was set at 100%. **c** Endogenous generation of AMP in culture supernatants of CD28WTCD73WTCD8$^{+}$ (n = 7) and CD28WTCD73KOCD8$^{+}$ (n = 5) T cells after stimulation by anti-CD3 (*black*) or anti-CD3/28 (*red*) antibodies was quantified. **d** The ability of CD8$^{+}$ T cells to degrade exogenous AMP to adenosine was analyzed. Exogenous AMP$_{13C,15N}$ (37.5 μM) was added to different cultures as indicated on the graph. Medium indicates the cultures did not have exogenous AMP$_{13C,15N}$ added. Supernatants were collected after incubation for 2 h. Adenosine$_{13C, 15N}$ metabolite was

quantified at the indicated retention time (x-axis). The quantity of adenosine$_{13C,\ 15N}$ in the supernatant of CD28WTCD73WTCD8$^{+}$ T cell culture with exogenous AMP$_{13C,15N}$ added and stimulated with anti-CD3 antibody was set at 100%. **e** Residual metabolite of AMP$_{13C,15N}$ isotope in the culture supernatants of CD8$^{+}$ T cells as reported in Fig. 4j after stimulation by anti-CD3 (*black*) or anti-CD3/28 (*red*) antibodies was quantified. Before 2h of measurement, these activated CD28WTCD73WTCD8$^{+}$ T cells were pretreated with: medium (n = 7), exogenous AMP$_{13C,15N}$ (n = 7) or exogenous AMP$_{13C,15N}$ plus iCD73 (n = 5), and the activated CD28WTCD73KOCD8$^{+}$ T cells were pretreated with medium (n = 5) or exogenous AMP$_{13C,15N}$ (n = 5). Statistical evaluations were performed using the Student's*t*-test with data expressed as the mean ± standard error of the mean (c, e). *$p < 0.05$, **$p < 0.01$; ns, not statistically significant.

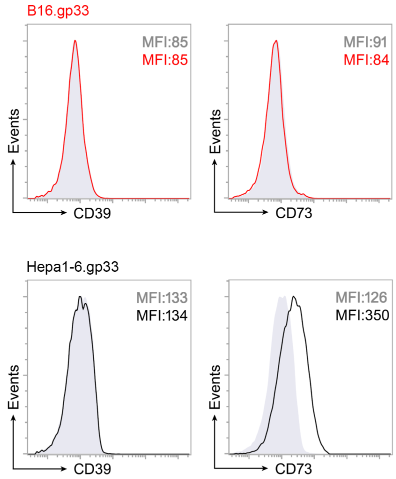


**Supplementary Figure 3. Murine melanoma B16.gp33 tumor cells do not express CD39 and CD73.** Hepa 1-6.gp33 cells (*black line*) and B16.gp33 cells (*red line*) were stained by PE-anti-mouse CD39 and PerCP-Cy5.5 CD73 antibodies and subjected to flow cytometric analysis. Hepa 1-6.gp33 cells were used as positive staining control for CD73. Hepa 1-6.gp33 cells and B16.gp33 cells were also stained by PE rat IgG2aκ or PerCP-Cy5.5 rat IgG1κ as isotype controls (*grey shadow*). Number on the upper right corner is the MFI of each individual stain. Data shown represent one of the three independent experiments.

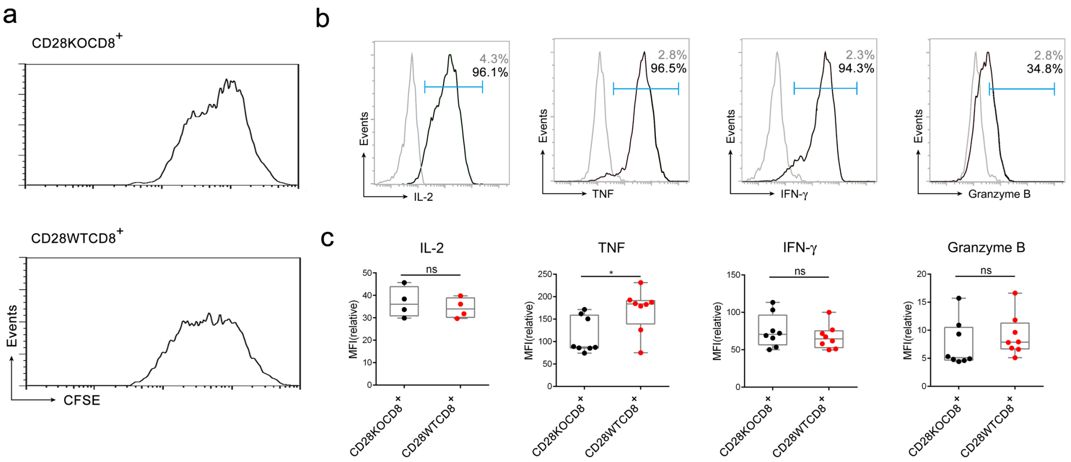


**Supplementary Figure 4. The effects of CD28 costimulation on CD8$^+$ T cell proliferation, cytokine and granzyme B production. a** Naïve CD8$^+$ T cells (3 × 10$^6$/mL) isolated from CD28KO and CD28WT mouse spleens were labeled with CFSE and stimulated with anti-CD3/28 antibodies. Cells were harvested at 64 h after stimulation and subjected to flow cytometric analysis of cell proliferation. *Upper panel*, CD28KOCD8$^+$ T cells. *Lower panel*, CD28WTCD8$^+$ T cells. Data presented are representative of one of the three independent experiments. **b** On day 5 after stimulation, CD28KOCD8$^+$ T cells were restimulated with PMA and ionomycin and subjected to intracellular staining of IL-2, TNF, IFN-γ and granzyme B and flow cytometric analysis. Percentages of cytokine- or granzyme B- producing populations of the activated CD28KOCD8$^+$ T cells (*black*) are shown. *Grey*: isotype control. Data presented are representative of one of the three independent experiments. **c** Naïve CD28KOCD8$^+$ and

CD28WTCD8$^+$ T cells were stimulated by anti-CD3/28 antibodies. On day 5 after stimulation, activated CD28KOCD8$^+$ T cells (*black*) and CD28WTCD8$^+$ T cells (*red*) were restimulated with PMA and ionomycin. Production of IL-2 (n = 4), TNF (n = 8), IFN-γ (n = 8) and granzyme B (n = 8) was determined by intracellular staining and analyzed by flow cytometry. Relative MFI is calculated by dividing the MFI of IL-2, TNF, IFN-γ and granzyme B stain by that of isotype control. Data were pooled from four independent experiments. Statistical evaluations were performed using the Student's*t*-test with data expressed as the mean ± standard error of the mean. *$p < 0.05$; ns, not statistically significant.

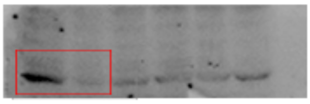

uncropped Figure 4d

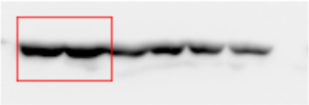

uncropped Figure 4d

**Supplementary Figure 5. Uncropped Western Blots.** Uncropped blots for Fig. 4d.
