# Supplementary material for: CD28 engagement inhibits CD73-mediated regulatory activity of CD8+ T cells
Source: Commun Biol. 2021 May 19;4:595. doi: 10.1038/s42003-021-02119-9 (PMC8134507; doi:10.1038/s42003-021-02119-9)
Supplement: Supplementary file 3 — Description of Additional Supplementary Files [file 42003_2021_2119_MOESM3_ESM.pdf]

Description of Additional Supplementary Files

File name: Supplementary Data 1

Description: All source data underlying the graphs and charts presented in the main figures.
