# Supplementary material for: CD28 engagement inhibits CD73-mediated regulatory activity of CD8+ T cells
Source: Commun Biol. 2021 May 19;4:595. doi: 10.1038/s42003-021-02119-9 (PMC8134507; doi:10.1038/s42003-021-02119-9)
Supplement: Supplementary file 5 — Reporting Summary [file 42003_2021_2119_MOESM5_ESM.pdf]

nature research

Corresponding author(s): Shu-Ching Chen

Last updated by author(s): Apr 5, 2021

Reporting Summary

Nature Research wishes to improve the reproducibility of the work that we publish. This form provides structure for consistency and transparency in reporting. For further information on Nature Research policies, see our Editorial Policies and the Editorial Policy Checklist.

Statistics

For all statistical analyses, confirm that the following items are present in the figure legend, table legend, main text, or Methods section.

n/a	Confirmed	
☐	☒	The exact sample size (*n*) for each experimental group/condition, given as a discrete number and unit of measurement
☐	☒	A statement on whether measurements were taken from distinct samples or whether the same sample was measured repeatedly
☐	☒	The statistical test(s) used AND whether they are one- or two-sided<br>*Only common tests should be described solely by name; describe more complex techniques in the Methods section.*
☐	☒	A description of all covariates tested
☐	☒	A description of any assumptions or corrections, such as tests of normality and adjustment for multiple comparisons
☐	☒	A full description of the statistical parameters including central tendency (e.g. means) or other basic estimates (e.g. regression coefficient) AND variation (e.g. standard deviation) or associated estimates of uncertainty (e.g. confidence intervals)
☐	☒	For null hypothesis testing, the test statistic (e.g. *F*, *t*, *r*) with confidence intervals, effect sizes, degrees of freedom and *P* value noted<br>*Give P values as exact values whenever suitable.*
☒	☐	For Bayesian analysis, information on the choice of priors and Markov chain Monte Carlo settings
☒	☐	For hierarchical and complex designs, identification of the appropriate level for tests and full reporting of outcomes
☒	☐	Estimates of effect sizes (e.g. Cohen's *d*, Pearson's *r*), indicating how they were calculated

Our web collection on statistics for biologists contains articles on many of the points above.

Software and code

Policy information about availability of computer code

Data collection	1. BD FACSVerse™ (BD Biosciences) was employed for cell acquisition. 2. xPONENT® software. 3.MassLynx version 4.1 and TargetLynx software
Data analysis	1. BD FACSuite™ software for data analysis. 2. MILLIPLEX® Analyst 5.1 Software. 3.MassLynx version 4.1 and TargetLynx software. 4. GraphPad Prism version 6 software. 5. SAS software version 9.4

For manuscripts utilizing custom algorithms or software that are central to the research but not yet described in published literature, software must be made available to editors and reviewers. We strongly encourage code deposition in a community repository (e.g. GitHub). See the Nature Research guidelines for submitting code & software for further information.

Data

Policy information about availability of data

All manuscripts must include a data availability statement. This statement should provide the following information, where applicable:

- Accession codes, unique identifiers, or web links for publicly available datasets
- A list of figures that have associated raw data
- A description of any restrictions on data availability

All data needed to evaluate the conclusions in the paper are present in the paper or the Supplementary Materials. The data used to generate and support this study will be available from corresponding author upon request.

Field-specific reporting

Please select the one below that is the best fit for your research. If you are not sure, read the appropriate sections before making your selection.

- [x] Life sciences
- [ ] Behavioural & social sciences
- [ ] Ecological, evolutionary & environmental sciences

For a reference copy of the document with all sections, see nature.com/documents/nr-reporting-summary-flat.pdf

Life sciences study design

All studies must disclose on these points even when the disclosure is negative.

Sample size	The sample size is determined based on statistics significance of each experiment.
Data exclusions	No data are excluded from the analysis.
Replication	All attempts to reproduce data were successful.
Randomization	n.a.
Blinding	n.a.

Reporting for specific materials, systems and methods

We require information from authors about some types of materials, experimental systems and methods used in many studies. Here, indicate whether each material, system or method listed is relevant to your study. If you are not sure if a list item applies to your research, read the appropriate section before selecting a response.

Materials & experimental systems

n/a	Involved in the study	
[ ]	[x]	Antibodies
[ ]	[x]	Eukaryotic cell lines
[x]	[ ]	Palaeontology and archaeology
[ ]	[x]	Animals and other organisms
[ ]	[x]	Human research participants
[x]	[ ]	Clinical data
[x]	[ ]	Dual use research of concern

Methods

n/a	Involved in the study	
[x]	[ ]	ChIP-seq
[ ]	[x]	Flow cytometry
[x]	[ ]	MRI-based neuroimaging

Antibodies

Antibodies used	Anti-mouse CD3 (clone 145-2C11) and anti-CD28 (clone PV-1) antibodies were prepared in our laboratory. Anti-mouse antibodies including Alexa 488 anti-Foxp3, -IFNgamma and -perforin , PE anti-CD25, -CD39, -CD101, -CTLA-4, -granzyme B and -ICOS and -IL-2, and PerCP-Cy5.5 anti-CD73 and APC anti-CD45.2, FITC anti-human CD8, PE anti-human CD28, APC anti-human CD39 and PE-Cy7 anti-human CD73 antibodies from eBioscience (San Diego, CA, USA); PE anti-TNF, -CCR6, -CD103, -Galectin-9 and -Helios antibodies from BioLegend (San Diego, CA, USA); PE anti-CD122, -CTLA-4, -LAG3 and -PD-1, APC anti-FR4 and PE-Cy7 anti-GITR antibodies from BD Bioscience (San Jose, CA, USA); Anti-CD73 antibody from Abcam (Cambridge, MA, USA)
Validation	All antibodies were validated by the manufactures.<br>1. Cross-linking of P-selectin glycoprotein ligand-1 induces death of activated T cells. (Blood. 2004 Nov 15;104(10):3233-42.)<br>2. CD4+ T Cell-Derived IL-2 Signals during Early Priming Advances Primary CD8+ T Cell Responses (PLoS One. 2009 Nov 10;4 (11):e7766. doi: 10.1371/journal.pone.0007766.)

Eukaryotic cell lines

Policy information about cell lines

Cell line source(s)	1. B16.gp33 cells were derived from B16 melanoma cells and genetically modified to express gene encoding amino acid 33-41 of glycoprotein from lymphocytic choriomeningitis virus (LCMV) (kindly provided by Dr. Hanspeter Pircher)<br>2. Hepa 1-6.gp33 cells were derived from Hepa 1-6 cells and genetically modified to express gene encoding amino acid 33-41 of glycoprotein from lymphocytic choriomeningitis virus (LCMV). (Hepa 1-6 cells were purchased from Bioresource Collection and Research Center.)
Authentication	1. B16.gp33 cells : Dr. Hanspeter Pircher. 2. Hepa 1-6 cells: Bioresource Collection and Research Center

Mycoplasma contamination	All the cells used were tested Mycoplasma negative.
Commonly misidentified lines (See ICLAC register)	n.a.

Animals and other organisms

Policy information about studies involving animals; ARRIVE guidelines recommended for reporting animal research

Laboratory animals	Male C57BL/6, and P14 TCR transgenic mice 33,34 (P14, originally obtained from Dr. J. Kung, Academia Sinica) at age of 6-8 weeks were obtained from animal center at National Taiwan University Hospital. CD28KO mice were acquired from the Jackson Laboratory. P14CD28KO mice were generated through crossing P14 mice to CD28KO mice. Animals were bred and housed in specific-pathogen-free conditions at the animal center at National Taiwan University Hospital, according to international guidelines on the care and use of laboratory animals. CD73KO mice were acquired from the Jackson Laboratory and bred and housed in specific-pathogen-free conditions at National Laboratory Animal Center, National Applied Research Laboratories, Taiwan.
Wild animals	n.a.
Field-collected samples	n.a.
Ethics oversight	All animal experiments were performed following the guideline of the Use of Laboratory Animals published by NTU and approved by Institutional Animal Care and Use Committee of College of Medicine and College of Public Health of NTU and were performed in compliance with the 3R principle (IACUC #20100131).

Note that full information on the approval of the study protocol must also be provided in the manuscript.

Human research participants

Policy information about studies involving human research participants

Population characteristics	The age of all human research participants were between 20 to 80 years old. Blood and primary tumor samples were obtained from patients with non-pretreated colon cancer at National Taiwan University Hospital.
Recruitment	All human research participants were recruited voluntarily from National Taiwan University Hospital.
Ethics oversight	All of these samples were obtained after approval from the Institutional research ethics committee of National Taiwan University Hospital (protocol number: 202009044RINA, 12th October, 2020) before its commencement and patients' written informed consent.

Note that full information on the approval of the study protocol must also be provided in the manuscript.

Flow Cytometry

Plots

Confirm that:

- [x] The axis labels state the marker and fluorochrome used (e.g. CD4-FITC).
- [x] The axis scales are clearly visible. Include numbers along axes only for bottom left plot of group (a 'group' is an analysis of identical markers).
- [x] All plots are contour plots with outliers or pseudocolor plots.
- [x] A numerical value for number of cells or percentage (with statistics) is provided.

Methodology

Sample preparation	CD8+ T cells from spleens of mice were enriched by positive selection using magnetic bead kit (Miltenyi Biotec Inc., Bergisch Gladbach, Germany) prior to activation. CD62LhiCD44loCD8+ were sorted (FACSAria (BD Bioscience, San Jose, CA, USA) and used as naïve CD8+ T cells (purity ≥ 99%) throughout the experiment. through service provided by Flow Cytometric Analyzing and Sorting Core Facility (First Core Laboratory, NTU, College of Medicine). PBMCs were purified from blood of patients with non-pretreated colon cancer by Ficoll density gradient. TILs of colon cancer were obtained by dissociating tumors using GentleMACS mechanical system according to manufacturer's protocol.
Instrument	FACSAria and BD FACSVerse™ (BD Bioscience, San Jose, CA, USA)
Software	BD FACSuite™ software
Cell population abundance	Naïve mouse CD8+ T cells (CD62LhiCD44loCD8+, purity ≥ 99%)
Gating strategy	For detection of CD8+ T cells , cell population was gated based on size and granularity (SSC-A/FSC-A) and CD8 positive.

- [x] Tick this box to confirm that a figure exemplifying the gating strategy is provided in the Supplementary Information.
